# Niche construction and niche choice by aphids infesting wheat ears

**DOI:** 10.1007/s00442-024-05612-0

**Published:** 2024-09-03

**Authors:** Andreas Bühler, Rabea Schweiger

**Affiliations:** 1 Department of Chemical Ecology, Bielefeld University, Universitätsstraße 25, 33615 Bielefeld, Germany; 2 Joint Institute for Individualisation in a Changing Environment (JICE), University of Münster and Bielefeld University, Bielefeld, Germany

**Keywords:** Plant-aphid interaction, Ecological niche, Metabolites, Phloem, Bioassay

## Abstract

The niche of aphids is largely defined by their consumption of plant phloem sap and its composition, including nutrients and specialized metabolites. Niche construction is the change of the environment by organisms, which may influence the fitness of these organisms and their offspring. To better understand interactions between plants and aphids, it is necessary to investigate whether aphids modify the chemical composition of the phloem sap of their host plants and whether conspecifics are affected by previous infestation. In the current study, ears of wheat (*Triticum aestivum*) plants were infested with clonal lineages of the English grain aphid (*Sitobion avenae*) or were left uninfested. The metabolic composition of ear phloem sap exudates was analyzed through amino acid profiling and metabolic fingerprinting. Aphids of the clonal lineages were either put on previously aphid-infested or on uninfested ears and their colony sizes followed over time. Furthermore, it was investigated whether aphids choose one treatment group over another. *Sitobion avenae* infestation affected the relative concentrations of some metabolites in the phloem exudates of the ears. Compared to uninfested plants, the relative concentration of asparagine was higher after aphid infestation. Colonies grew significantly larger on previously aphid-infested ears, which the aphids also clearly chose in the choice experiment. The pronounced positive effect of previous infestation on aphid colonies indicates niche construction, while the choice of these constructed niches reveals niche choice by *S. avenae* on wheat. The interplay between these different niche realization processes highlights the complexity of interactions between aphids and their hosts.

**Supplementary Information:**

The online version contains supplementary material available at 10.1007/s00442-024-05612-0.

## Introduction

Plant–herbivore interactions are highly dynamic, as the interaction partners have numerous, varied effects on each other. In these interactions, the chemical composition of the host plant plays a crucial role, as herbivores rely on nutrients but may also be negatively affected by defensive compounds (Howe and Jander 2008). Thus, for herbivores the plant metabolome is an important part of their ecological niche, which describes the ranges of environmental factors allowing organisms to survive and reproduce (Devictor et al. 2010). The niche concept and feeding niches of herbivores are essential to understand plant–herbivore interactions. Herbivores may modify the chemical composition of their host plants, including an induction of plant defenses (Chen 2008; Howe and Schaller 2008; Mai et al. 2014) as well as having effects on primary metabolites (Zhou et al. 2015; Chiozza et al. 2010). A modification of the environment by organisms, which may affect their fitness, is referred to as niche construction (Müller et al. 2020; Odling-Smee et al. 2003; Trappes et al. 2022) and may also affect subsequent generations (Odling-Smee et al. 2003). Therefore, the modification of the host metabolism by herbivores through changing the composition of nutrients and defensive compounds in ways that influences their fitness and that of their offspring may be seen as niche construction.

Aphids are useful to study ecological niches including niche construction for two reasons. Because they almost exclusively feed on the phloem sap of their hosts, its chemical composition contributes largely to their ecological niche. Phloem sap is rich in sugars, but has a low concentration of amino acids (Douglas 2006; Lohaus 2022). Amino acids are vital for aphids and strongly influence their choices (Gruhn et al. 2021; Karley et al. 2002; Nowak and Komor 2010; Ponder et al. 2000). The phloem sap also contains specialized metabolites (Lohaus 2022; Turgeon and Wolf 2009), many of which are anti-herbivore defenses. For example, benzoxazinoids, which occur in the phloem sap of wheat (Givovich et al. 1994), negatively affect aphids (Maag et al. 2015; Wouters et al. 2016). Thus, chemical analyses of phloem sap/ exudates focussing on amino acids and specialized metabolites can provide essential understanding of aphids’ niche parameters. The second reason for the suitability of aphids for research on niches is that they reproduce parthenogenetically and rapidly (Guerrieri and Digilio 2008; Minks and Harrewijn 1987). Thus, lineages of genetically identical offspring can serve as proxies for individuals, allowing for comparisons of the performance of the same “individual” under different conditions.

There is strong evidence that aphids change the chemical composition of their host plants, with some of it hinting at niche construction. While aphids may activate the salicylic acid and jasmonic acid defense pathways (Zhao et al. 2009), aphid saliva contains protein effectors that may suppress plant defense (Will et al. 2007; Zhang et al. 2022). Aphid-induced changes of the concentrations of specialized metabolites such as benzoxazinoids (Niemeyer et al. 1989; Zhang et al. 2021) have mainly been demonstrated for plant leaves, while studies addressing aphid-induced changes of specialized metabolites in plant phloem sap/ exudates are rare (but see Jakobs et al. 2019). In studies specifically investigating phloem sap/ exudate for primary metabolites, a larger fraction of essential amino acids after aphid infestation could be shown for some plant-aphid systems (Liu et al. 2020; Sandström et al. 2000). Other studies have shown various effects of previous aphid infestation on the performance of aphids (Fisher 1987; Petersen and Sandström 2001; Qureshi and Michaud 2005; Takemoto et al. 2013). However, studies are needed to link aphid-induced metabolic changes as a potential mechanism of niche construction with effects on the fitness of the aphids.

*Sitobion avenae*, the English grain aphid, is a prevalent pest of cereals, including wheat (Larsson 2005; Vickerman and Wratten 1979). Studies of the interaction of *S. avenae* with *Triticum aestivum* have uncovered aphid-induced activation of defense pathways (Zhao et al. 2009) and a higher fraction of essential amino acids in phloem exudates of seedlings (Liu et al. 2020). However, in young wheat plants we found no effects of *S. avenae* on the composition of leaf phloem exudates and only a transient increase of aphid performance on aphid-infested leaves (Bühler and Schweiger 2024). Because ears seem to be the preferred niche of this aphid species, the question was raised whether niche construction can be found in ears. Indeed, *S. avenae* migrates to the ears as soon as they emerge and is mainly found and better performs on the ears and flag leaves than on older leaves of cereals (Honek et al. 2017; Watt 1979; Wratten 1975). Moreover, *S. avenae* was faster in choosing between ears than between seedlings of wheat varieties (De Zutter et al. 2012). This indicates that *S. avenae* is a specialist not just for cereals, but specifically adapted to and choosing the ears, highlighting the need to explore its niche construction ability on these plant parts. As the metabolic composition of wheat phloem exudates differs between leaves and ears (Stallmann et al. 2022), it is important to analyze the phloem exudates of ears in studies on the niche construction ability of *S. avenae* on these plant parts.

To investigate whether there is niche construction by *S. avenae* on wheat ears, the chemical composition of phloem exudates was compared between ears that were uninfested or infested by *S. avenae*, assessing amino acids and putative specialized metabolites. Moreover, the colony sizes on previously uninfested ears and on ears that had been pre-infested by a different or by the same clonal lineage were compared and choices of aphids between these ears were tested. We expected infestation by *S. avenae* to change the composition of amino acids, including a larger fraction of essential amino acids, and to affect the metabolic fingerprint of the phloem exudates. Aphid colony sizes were supposed to benefit from previous aphid infestation. Furthermore, we expected *S. avenae* to choose previously aphid-infested ears. We expected stronger effects of a previous infestation on aphid performance and preference if the pre-infesting aphids were from the same clonal lineage, as niche construction may differ between aphid genotypes and may have evolved under the selection pressure of fitting to the probably aphid genotype-specific niche requirements.

## Materials and methods

**Fig. 1:**
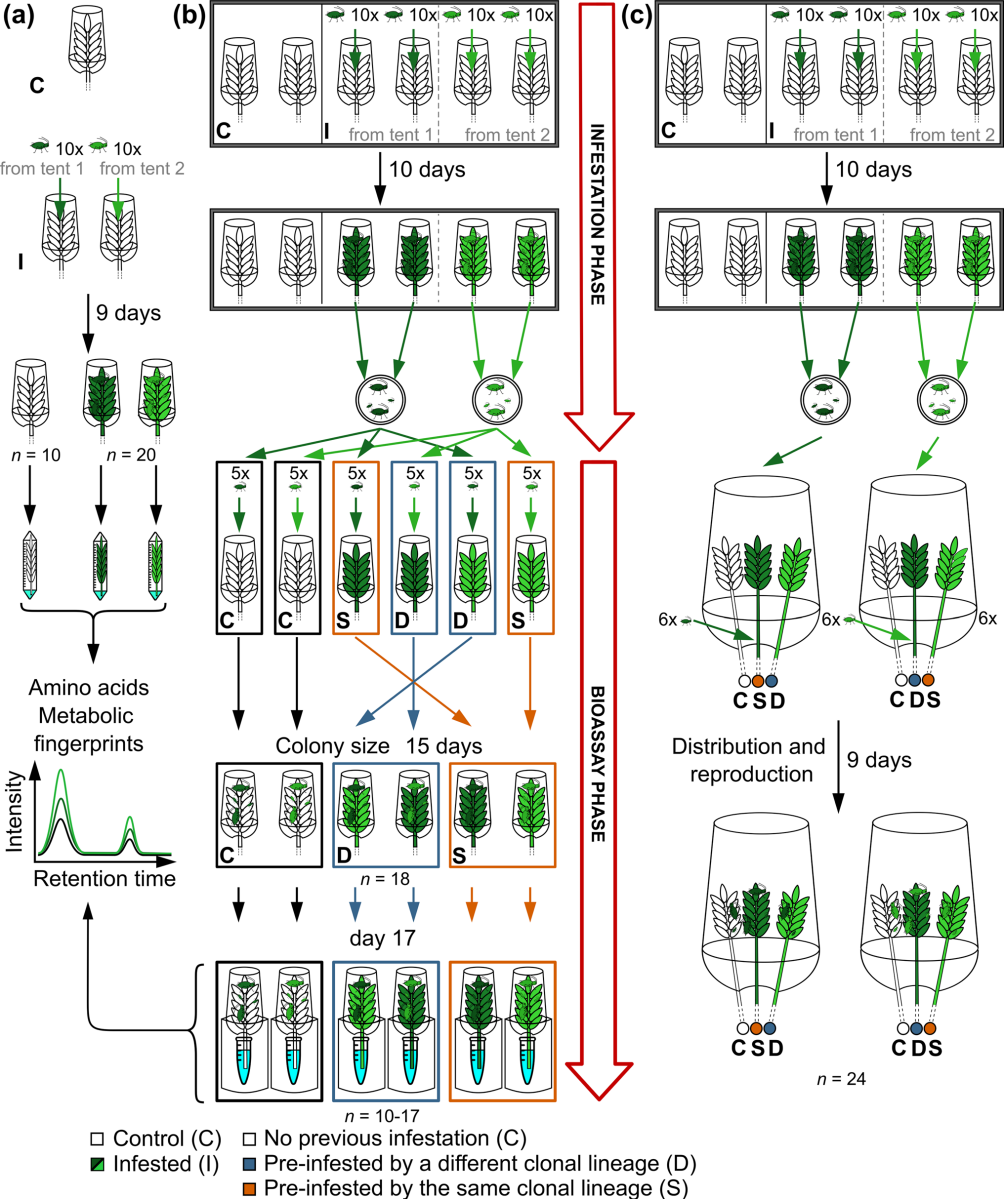
Setups for different experiments. For each of the three experiments, a separate batch of plants was used. Ears of *Triticum aestivum* plants were left uninfested (control plants, C) or infested by *Sitobion avenae* (I) during the infestation phase. **a** Phloem exudates were collected at the end of the infestation phase as well as at the end of the bioassay phase of the performance bioassay (see below) and analyzed for their metabolic composition. **b**, **c** Two bioassays were performed, for which nymphs were redistributed after the infestation phases for the following bioassay phases. Bioassays were done using ears of intact plants; for simplicity, only the ears are shown and the remaining plant parts are indicated by hatched lines. In the performance bioassay (**b**), the nymphs were placed on previously uninfested ears (white), on ears pre-infested by a different clonal lineage (D, blue) or on ears pre-infested by the same clonal lineage (S, orange). The colony sizes of the aphids were followed over time. In the choice bioassay (**c**), the choices of aphids between ears of plants of the three treatment groups were assessed. The colours of the ears correspond to their treatment in the infestation phase (white: C; dark green: aphid lineage from tent 1; light green: aphid lineage from tent 2). Sample sizes are indicated at the bottom

To investigate effects of an infestation with *Sitobion avenae* on the chemical composition of phloem exudates of wheat ears and on conspecific aphids, three experiments with three separate batches of plants were conducted (Fig. 1), similar as done for wheat leaves (Bühler and Schweiger 2024). Randomized block designs were applied. Experiments were done in climate chambers (16:8 h light:dark, 20 °C, 70% relative humidity).

### Plants

Seeds of *Triticum aestivum* L. (Poaceae; spring wheat variety Tybalt; von Borries-Eckendorf, Leopoldshöhe, Germany) were germinated in a mixture of river sand: soil (Fruhstorfer Spezialsubstrat Type P; Hawita Group, Vechta, Germany) of 2:1 (v:v), steamed at 90–95 °C. Seven days later, plants were transferred to 2 l pots (11.3 cm by 11.3 cm, height 21.5 cm) containing the same substrate. Each plant was fertilized twice with 1.5 g fertilizer (Plantosan N-P_2_O_5_-K_2_O 20–10–15, 6% MgO, 2% S, traces of B, Cu, Fe, Mn, Mo and Zn; Manna, Düsseldorf, Germany) 34 and 50 days after sowing. Thrice a week, plants were watered with equal amounts of tap water, with amounts increasing over time.

### Aphids

*Sitobion avenae* (Fabricus; Aphididae) aphids were from re-natur (Stolpe, Germany) and reared in two tents (50 cm by 50 cm) on 10–20-day old, non-flowering wheat (cv. Tybalt) using the substrate described above. Aphids reproduced parthenogenetically, producing only apterous females. To allow testing individuals with the same genetic background under different conditions, monoclonal aphid lineages were created by putting single adult, apterous females into gauze cages (diameter 20 cm, height 40 cm) with 2-week-old wheat seedlings and allowing them to reproduce. Each monoclonal lineage represented one replicate. The clonal lineages probably covered different aphid genotypes, as their founding aphids were taken from different plants and from the two tents, for which aphids were purchased at dates several months apart. For the comparison of previous aphid infestation on aphids of a different clonal lineage compared to those of the same clonal lineage (see bioassays below), the lineages that were compared originated from different tents to increase the probability that the genotypes differed. For the experiments, aphids were age-standardized by placing 60 adults of each lineage in a cage, removing them after 48 h and leaving the nymphs.

### Experiment (a): Phloem exudates

One batch of plants was used to collect phloem exudates of ears of control (C) plants and of aphid-infested (I) plants (Fig. 1a). Fifty-seven days after sowing, the ears of the main stems of the plants were equipped with cages (PET cups with lids; WIMEX s.r.o, Náchod, Czech Republic; diameter 95 mm, 500 ml, partly replaced by a fine metal mesh). The wheat ears were inserted through cuttings in white foam rubber. The plants of the infestation group (I) were treated with ten apterous females (13 to 15 days old) of a given clonal lineage by putting the aphids into the cages next to the ear stalks with a fine brush. The cages and stalks of control (C) plants were touched with a brush for a similar amount of time. After 9 days, phloem exudates of the ears were collected using ethylenediaminetetraacetic acid (King and Zeevaart 1974) as described earlier (Stallmann et al. 2022) with modifications. Aphids were removed from the infested plants (and counted later on), while the ears of control plants were brushed for a similar time. Then, the ears were cut off with a razor blade at least 3 cm outside the cages. The cut edges were submerged and re-cut in 8 mM ethylenediaminetetraacetic acid solution (99%, AppliChem, Darmstadt, Germany; pH = 7, adjusted with NaOH) and put in 1 ml ethylenediaminetetraacetic acid solution in 50 ml Falcon tubes with a second Falcon tube as a lid. The Falcon tubes were placed in the dark for 2 h, the cut edges were washed with Millipore water and transferred to fresh tubes containing 1 ml Millipore water and incubated for another 2 h. Then, 400 µl subsamples of the phloem exudates were shock-frozen in liquid N_2_ and stored at  – 80 °C. Blanks were prepared from Millipore water kept in the tubes without plant material. Phloem exudates were freeze-dried and redissolved in 50 µl 85% methanol (v:v; LC–MS grade; Thermo Fisher Scientific, Loughborough, UK) with the internal standards L-norvaline and sarcosine (Agilent Technologies, Waldbronn, Germany) for primary and secondary amino acids, respectively, and hydrocortisone (Sigma-Aldrich, Steinheim, Germany) for metabolic fingerprinting. Amino acids were analyzed using high pressure liquid chromatography coupled to fluorescence detection modified from Jakobs and Müller (2018), while metabolic fingerprinting was accomplished with ultra-high performance liquid chromatography coupled to quadrupole time-of-flight mass spectrometry modified from Schweiger et al. (2021) (Supplementary Methods 1 in Online Resource 1). Metabolic fingerprinting is a non-targeted comparative approach to assess metabolic differences between experimental conditions.

### Experiment (b): Aphid performance bioassay

The influence of previous aphid infestation on aphid performance was assessed using a second batch of plants (Fig. 1b). As described in detail for Experiment (a), 57 days post sowing the ears of the main stems of the plants were equipped with cages, ears of the infestation group (I) were infested with aphids (13 to 15 days old) and control plants (C) were touched with a brush for a similar amount of time. After 10 days, aphids from the clonal lineages from tent 1 and tent 2 were separately collected (and counted later on), touching control plants with a brush for a similar amount of time. For each clonal lineage, 15 mid-instar nymphs were selected and five nymphs were applied to each cage (C and I) on the foam rubber close to the ear stalk, using new cages. Nymphs were chosen to test for ecological inheritance, i. e. effects of aphid infestation on the next generation; the mid-instar stage allowed handling the nymphs without damaging them. After the redistribution of the nymphs, in the bioassay phase the ears were either previously uninfested (C), pre-infested by conspecifics of a clonal lineage from the other tent (different lineage, D) or by the same lineage (S). The sizes of the aphid colonies (i. e. number of surviving aphids) were assessed for 15 days. At day 17, phloem exudates were collected as described above, but leaving the cages and aphids on the plants and using 2 mL Eppendorf tubes sealed with Parafilm (Fig. 1a). The foam rubber at the bottom of the cages prevented a contamination of the phloem exudate collection solution with aphid honeydew.

### Experiment (c): Aphid choice bioassay

The influence of previous aphid infestation on the choices of aphids was assessed using a third batch of plants (Fig. 1c). The treatments and collection of aphids after the infestation phase were similar as for the aphid performance bioassay (see above; C, control; I, infested with 12- to 14-day-old aphids at 64 days post sowing), while in the bioassay phase six mid-instar nymphs were put into choice cages containing three ears each. The aphids were put on the foam rubber between the stalks of the ears; to reach the ears, they needed to overcome circa 3 cm distance to the stalks of the ears and to climb up 5 cm. The ears were either previously uninfested (C), pre-infested by conspecifics of a clonal lineage from the other tent (different lineage, D) or by the same lineage (S). The number of founding aphids on the different ears as well as the number of their nymphs was assessed after one hour, after one day and then up to 9 days. These time points were chosen to record the early choice of the aphids but to also account for later redistributions of them. After 9 days, nymphs reached adulthood and could no longer be distinguished from their mothers, thus the experiment was terminated.

### Data analyses

Amino acids and metabolic features (characterized by specific RT and *m*/*z*) were identified and quantified as described in Supplementary Methods 1 (Online Resource 1). The following amino acids were categorized as essential for aphids based on Douglas (2006): histidine, threonine, valine, methionine, tryptophan, phenylalanine, isoleucine, leucine and lysine. As the ears of the treatment groups may have had different phloem exudation rates, relative concentrations (%) instead of absolute concentrations of metabolites were used. For the phloem exudates collected at the end of the performance bioassay, the sample size was reduced to *n* = 10–17, as some samples, possibly due to the age of the plants or problems during the phloem exudate collection, did not contain sufficient metabolites.

All further data analyses were performed in R 3.6.1 (R Core Team 2019) using the packages *car* (Fox and Weisberg 2019), *rstatix* (Kassambara 2021) as well as (for violin plots with embedded box-and-whisker diagrams) the functions ggplot, geom_violin and geom_boxplot within the package *ggplot2* (Wickham 2016). Data of the different treatment groups at the end of the infestation phase (C, control; I, infested) and in the bioassays (C, no previous infestation; D, pre-infested by a different clonal lineage; S, pre-infested by the same clonal lineage), respectively, were compared. Normal distributions were tested using Shapiro–Wilk tests, homoscedasticity was assessed using Levene tests and (for paired data) Mauchly tests were applied to test sphericity. α = 0.05 was used as significance threshold for all tests, with α = 0.10 as threshold for marginal significance. The fraction of essential amino acids and the relative concentrations of amino acids and metabolic features were compared between the groups (at end of infestation phase: I versus C; at end of performance bioassay: D versus C, S versus C) using Mann–Whitney-*U*-tests with Bonferroni-Holm correction. For the relative concentrations of metabolites, fold changes were calculated as the ratios of the means of the relative concentrations in the two groups (I/C, D/C, S/C). An amino acid or a metabolic feature was considered as being “modulated” by aphid infestation (I versus C; at end of infestation phase) and as being “differential” between groups (D versus C, S versus C; at end of performance bioassay), respectively, if its relative concentrations significantly differed between the groups and if it only occurred in one group (in at least half of the replicates) or occurred in both but with a fold change of < 0.5 (< -1 on log_2_ scale) or > 2 (> 1 on log_2_ scale). For the bioassays, statistical tests accounting for paired data were applied, as aphids from the same clonal lineage were tested on plants of different infestation treatment groups, testing time points separately. The colony sizes (different time points) of the performance bioassay and the proportions of surviving founding aphids and the number of their nymphs (different time points) in the choice bioassay were compared between the groups (C, D, S) using Friedman tests followed by pairwise Wilcoxon signed-rank tests, applying Bonferroni-Holm correction. For the choice bioassay, only aphids found on ears were included.

## Results

### Amino acids and metabolic features in phloem exudates

**Fig. 2:**
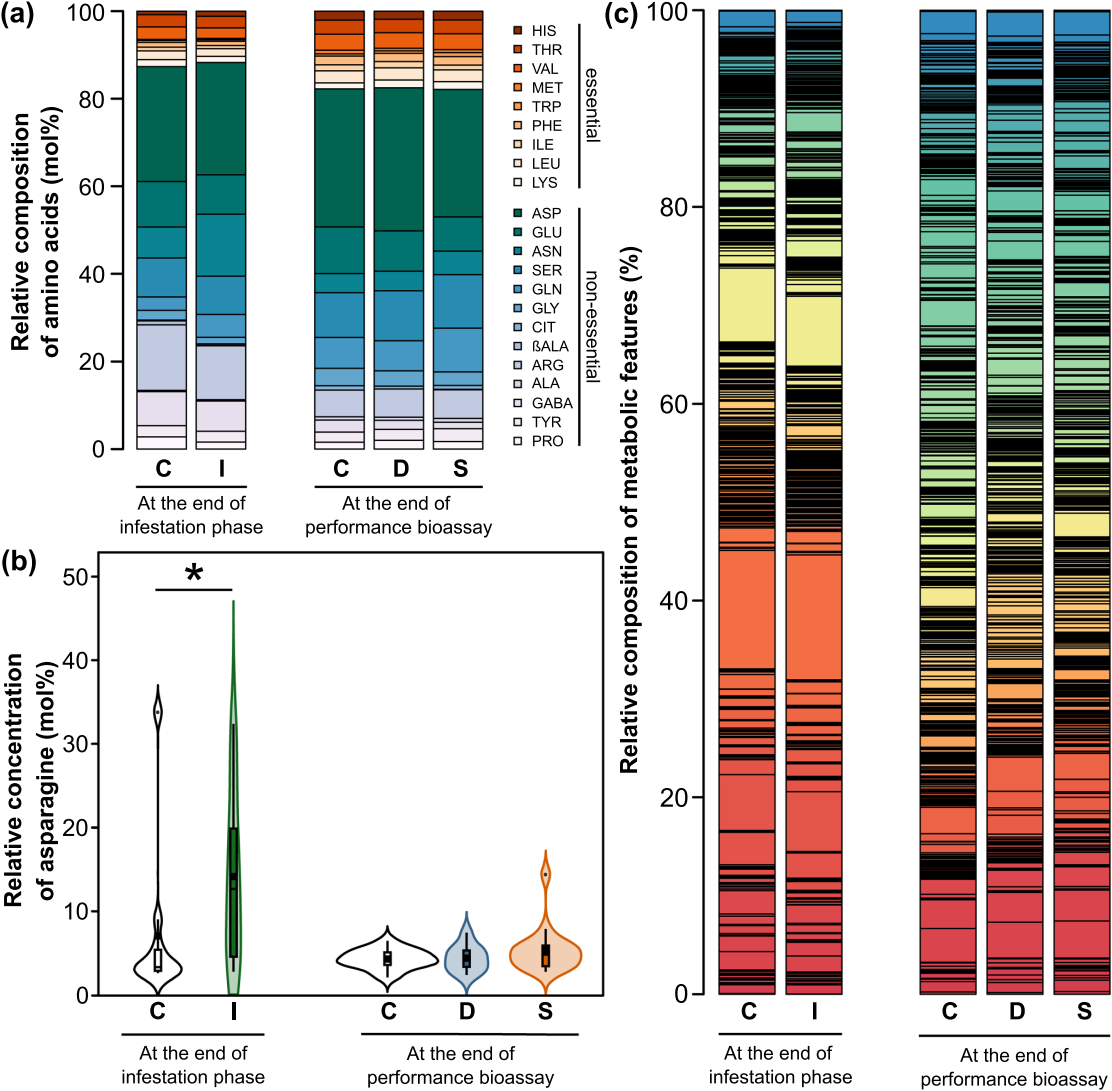
**a** Relative composition of amino acids, **b** relative concentration of asparagine and **c** relative composition of metabolic features in phloem exudates of ears of *Triticum aestivum*. Within each sub-graph, the data on the left belong to sampling at the end of the infestation phase, in which ears were either uninfested (C, *n* = 10 biological replicates) or infested by *Sitobion avenae* aphids (I, *n* = 20). The data on the right correspond to samples taken at the end of the performance bioassay, i. e. after previously uninfested ears had been infested by aphids (C, *n* = 16) or after pre-infested ears had been infested by a different clonal lineage (D, *n* = 10) or by the same clonal lineage (S, *n* = 17) for 17 days. **a**, **c** Mean relative concentrations per treatment group as percentage, shown as stacked bar plots. The amino acids (**a**) are distinguished into essential (reddish scale) and non-essential (bluish/ greenish scale) ones, data are given as mol% and full names and further data can be found in Tables S1 and S2 in Online Resource 1. **b** Relative concentration of asparagine, shown as violin plots. The widths of the violin plots represent the kernel probability densities for the different asparagine concentrations, while the violin areas are scaled proportionally to the sample sizes. In the embedded box-and-whisker diagrams, interquartile ranges (IQR) are shown as boxes, medians as thick horizontal lines, means as large squares, whiskers extend from the box to the most extreme data points with maximum 1.5 times the IQR and small circles represent outliers. The significant difference between the infestation treatment groups is shown (Mann–Whitney *U*-test; *P < 0.05), while non-significant differences are not indicated

We found 22 amino acids (Fig. 2). On average per treatment group, aspartic acid was the most common amino acid in all groups and made up between 25.7% and 32.7% on a molar basis (Fig. 2a). In samples taken at the end of the infestation phase, this was followed by arginine in control plants and by asparagine in aphid-infested plants, while at the end of the performance bioassay the second most common amino acid was either glutamic acid or serine. Averaged across groups, essential amino acids made up between 11.7% and 12.6% at the end of the infestation phase and 17.5% to 17.9% at the end of the performance bioassay. The fraction of essential amino acids did not differ significantly between groups at the end of the infestation phase (Mann–Whitney *U*-test: *W* = 83; *P* = 1) or at the end of the performance bioassay (D versus C: Mann–Whitney *U*-test, *W* = 78, *P* = 1; S versus C: Mann–Whitney *U*-test, *W* = 121, *P* = 1). The composition of amino acids was similar between the groups both at the end of the infestation phase and at the end of the performance bioassay (Fig. 2a). Only asparagine showed differences in its relative concentration between the groups (Tables S1 and S2 in Online Resource 1, Fig. 2b). At the end of the infestation phase, this amino acid was modulated by the aphid treatment. The phloem exudates of ears that had been infested by aphids had higher relative concentrations of asparagine than those of ears from control plants (log_2_ fold change 1.01; Mann–Whitney *U*-test: *W* = 42, *P* = 0.011). At the end of the performance bioassay, no amino acids were differential between groups.

The metabolic fingerprinting dataset contained 739 metabolic features (Fig. 2c, Online Resource 2). Of these, 74 were only detected in phloem exudates collected at the end of the infestation phase, while 19 only occurred in samples collected at the end of the performance bioassay. The treatment groups showed similar metabolic fingerprints (Fig. 2c). At the end of the infestation phase, five features were modulated by aphid infestation (I versus C), while at the end of the performance bioassay four (D versus C) and seven (S versus C) features were differential between the groups. Of these features, one was differential between both the D and S treatment groups and the control group, while all other features were only differential in one comparison. Two compounds could be putatively identified: phenylalanine (RT 1.99 min, *m*/*z* 166.0858) and tricin (4′,5,7-trihydroxy-3′,5′-dimethoxyflavone; RT 20.30 min, *m*/*z* 331.0806), both showing no differences between the groups.

### Aphid performance

**Fig. 3:**
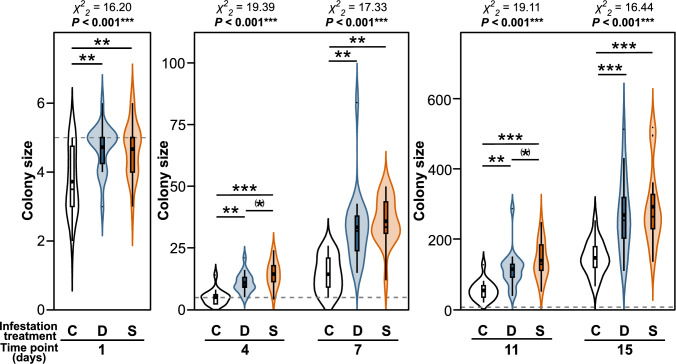
Effects of aphid infestation on the colony sizes of *Sitobion avenae* aphids over a period of 15 days during the performance bioassay. Aphids were on ears of *Triticum aestivum* plants, which were either previously uninfested (C, white) or pre-infested by a different clonal lineage (D, blue) or by the same clonal lineage (S, orange). The initial colony size at the beginning of the bioassay phase is given in form of hatched horizontal lines. Data are shown for different time points after the beginning of the bioassay, using violin plots. The widths of the violin plots represent the kernel probability densities for the different colony sizes. In the embedded box-and-whisker diagrams, interquartile ranges (IQR) are shown as boxes, medians as thick horizontal lines, means as large squares, whiskers extend from the box to the most extreme data points with maximum 1.5 times the IQR and small circles represent outliers. Different y-axis scaling was used for early and late time points, because colonies grew much over time. For each time point, results of Friedman tests (*χ*^*2*^-values, *P-*values) are shown above the graphs. Significant differences between the groups are indicated above the plots (Wilcoxon signed-rank tests; non-significant differences not indicated). (*) marginally significant at *P* < 0.1, **P < 0.01, ***P < 0.001, *n* = 18 biological replicates

In the performance bioassay, aphid colonies grew larger on previously aphid-infested than on previously uninfested ears (Fig. 3). This was found for the case that the pre-infesting aphids were from a different clonal lineage as well as for the case that they were from the same clonal lineage as the aphids of the bioassay phase. The bigger colonies on ears pre-infested by aphids were found throughout the experiment at different time points. The colony sizes were similar between the ears that had been pre-infested by a different clonal lineage (D) and those that had been pre-infested by the same clonal lineage (S), but there were slight tendencies (marginally significant at 4 days, 11 days) for higher colony sizes on ears that had been pre-infested by the same clonal lineage from the third day onwards.

### Aphid choices

**Fig. 4:**
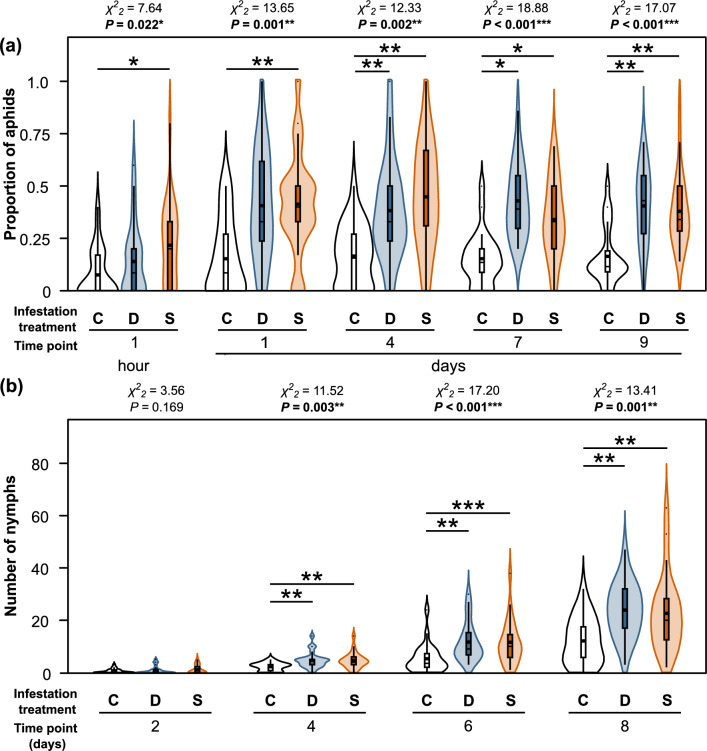
Effects of aphid infestation on the **a** proportions of *Sitobion avenae* aphids and **b** number of their nymphs over a period of 9 days during the choice bioassay. Aphids were positioned on ears of *Triticum aestivum* plants, which were either previously uninfested (C, white) or pre-infested by a different clonal lineage (D, blue) or by the same clonal lineage (S, orange). Aphids not positioned on the ears are not shown. Data are shown for different time points, using violin plots. The widths of the violin plots represent the kernel probability densities for the different proportions of aphids (**a**) and numbers of nymphs (**b**), respectively. In the embedded box-and-whisker diagrams, interquartile ranges (IQR) are shown as boxes, medians as thick horizontal lines, means as large squares, whiskers extend from the box to the most extreme data points with maximum 1.5 times the IQR and small circles represent outliers. For each time point, results of Friedman tests (*χ*^*2*^-values, *P*-values) are shown above the graphs. Significant differences between the groups are indicated above the plots (Wilcoxon signed-rank tests; non-significant differences not indicated). For the statistical tests, only aphids that were found on the ears were included. *P < 0.05, **P < 0.01, ***P < 0.001. *n* = 24 biological replicates

In the choice bioassay, one hour after the beginning of the bioassay many aphids had not yet moved to any ears, though of those that had, significantly more were found on ears pre-infested by the same clonal lineage compared to previously uninfested ears (Fig. 4a). At all following time points, many more aphids were found on the ears and aphids were significantly more likely found on ears that had been pre-infested by aphids of a different or the same lineage than on previously uninfested ears. When comparing the aphid choices between ears that had been pre-infested (S versus D), at the second day (marginally significant) more aphids were found on the ears that had been pre-infested by the same clonal lineage, while at the other time points there were no significant differences and no consistent tendencies. The first nymphs were produced two days after the beginning of the bioassay (Fig. 4b). At all later time points, significantly more nymphs were found on pre-infested ears, independent on which clonal lineage had previously infested them, with no significant differences between the S and the D group and no consistent tendencies for the comparison of these groups.

## Discussion

To study potential niche construction and niche choice by *Sitobion avenae* on wheat ears and potential underlying mechanisms at the metabolic level, we investigated the effects of an infestation by *S. avenae* on the phloem exudates of wheat ears and of a previous aphid infestation on the performance and choice of conspecifics.

The amino acid profiles of the wheat ears were made up mostly of non-essential amino acids, especially aspartic acid, arginine, asparagine, glutamic acid and serine. Likewise, non-essential amino acids dominated in phloem exudates of wheat ears in Stallmann et al. (2022). In Palmer et al. (2014), the phloem sap of wheat ears obtained by aphid stylectomy showed high concentrations of glutamine, valine, histidine and serine. Differences in amino acid profiles between studies may partly be explained by methodology, i. e. collection of phloem exudates versus phloem sap. Moreover, the relative concentrations of amino acids in phloem exudates of wheat ears vary both during the day and with ear development (Palmer et al. 2014; Palmer and Stangoulis 2018). Various putative specialized metabolites were detected in the phloem exudates of the wheat ears, including the flavonoid tricin. In wheat, tricin is mainly found in the inflorescences and husks (Moheb et al. 2013a, b). Tricin and related compounds may affect the fecundity of *Acyrthosiphon pisum* aphids (Goławska et al. 2012) and tricin is an antifeedant against *Myzus persicae* aphids (Dreyer and Jones 1981).

At the end of the infestation phase, the phloem exudates of aphid-infested wheat ears showed higher relative concentrations of asparagine. In plants, asparagine and glutamine are used for nitrogen transport related to protein degradation and senescence (Lea et al. 2007; Sieciechowicz et al. 1988). In cereals, during grain filling these metabolites are transported from the leaves to the grains (Masclaux et al. 2001). Asparagine is also associated with drought and temperature stress in plants, including wheat (Hale et al. 2003; Lea et al. 2007; Oddy et al. 2020; Xie et al. 2020). The higher relative concentration of asparagine in phloem exudates of previously aphid-infested ears may therefore be a stress response, mobilizing nitrogen to fill the grains for reproduction. Indeed, aphid infestation increased the senescence of leaves, including a loss of chlorophyll and higher concentrations of amino acids (Dillwith et al. 1991; Dorschner et al. 1987), which are probably beneficial for aphids. However, there was no visual difference in the senescence of leaves or ears between the treatment groups in our experiment. Thus, the higher relative concentration of asparagine in pre-infested ears may indicate an early stage of senescence. Infestation by *S. avenae* likewise affected several putative specialized metabolites in the phloem exudates. Some of these changes may have been caused indirectly, through the deposition of honeydew or the transfer of plant viruses by the aphids, which both may affect plant metabolites. Alternatively, metabolic changes may be due to plant signalling pathways elicited after perception of cues from the aphids or due to effector proteins in the aphid saliva. Effector proteins downregulate phytohormone-associated defense genes and change the phenolic profiles of plants (Rodriguez and Bos 2013; Urbanska et al. 1998; Zhang et al. 2022). Previous studies rarely investigated the influence of aphid infestation on specialized metabolites in the phloem sap of plants. However, in Jakobs et al. (2019) 3-caffeoyl-quinic acid in phloem exudates of *Tanacetum vulgare* was slightly affected by aphids in stems and young leaves and only occurred in phloem exudates of previously aphid-infested but not in uninfested old leaves.

We found clear evidence for niche construction by *S. avenae*, as indicated by the larger aphid colonies on previously aphid-infested ears. The positive effect of a previous infestation by *S. avenae* on conspecifics may at least partly be linked to aphid-induced metabolic changes. Specifically, the aphids may have profited from an aphid-induced senescence, transport of nitrogen to the grains, and/ or from suppression of plant defense (see Discussion above). The ripening stage of ears of cereals influences the performance of *S. avenae* (Watt 1979). Thus, a change in the development of the ear due to mobilization of amino acids from other plant parts could explain the results. The observed effects of pre-infestation by aphids may also be due to changes in absolute concentrations of metabolites, which could not be assessed with the phloem exudate collection method we used. For example, a higher aphid performance could be the result of lower concentrations of defensive metabolites or of higher concentrations of total or individual (e.g. essential) amino acids. The higher proportion of asparagine in aphid-infested ears may have positively affected aphid performance; to the best of our knowledge, effects of this individual amino acid on *S. avenae* have not been investigated/ reported before. Alternatively or in addition, the assumed aphid-induced N remobilization could have led to higher concentrations of amino acids in general, which could have benefited the aphids. An increase in the total amino acid concentration in ears was found as a result of *S. avenae* infestation on triticale (Sempruch et al. 2011). In contrast to others (Liu et al. 2020; Sandström et al. 2000; Telang et al. 1999), we did not find higher fractions of essential amino acids after aphid infestation that could explain the better aphid performance.

There is evidence that the ability of *S. avenae* to construct a niche depends on the age of the plants and/ or on the plant part of wheat. For wheat leaves, there was no influence of *S. avenae* on the composition of phloem exudates and positive effects on aphid performance were only transient (Bühler and Schweiger 2024). Together with the current study, this indicates that the niche construction ability by *S. avenae* on wheat is mainly restricted to its preferred niche, i. e. the ears (De Zutter et al. 2012; Honek et al. 2017; Watt 1979; Wratten 1975). The preference of *S. avenae* for ears may partially be explained by differences in the phloem exudate metabolome between ears and leaves of wheat (Stallmann et al. 2022) and the fact that the grains act as a nitrogen sink (Thorne 1985), likely reflecting a higher nutritional quality of the phloem sap of ears for the aphids. The quality of plant phloem sap is considered to be a main factor influencing aphid performance (Nalam et al. 2021).

*Sitobion avenae* showed a clear preference for wheat ears that had previously been infested by conspecifics. This shows that the aphids are able to recognize a previously infested host and choose a niche offering greater fitness (see above). Likewise, *Rhopalosiphum padi* aphids avoided wheat plants pre-infested by conspecifics, on which their fitness was lower (Messina et al. 2002). Thus, aphids may choose their feeding sites based on their expected fitness. The choice of the aphids may be due to cues perceived along their stylet path, where cells are punctured, or to clues within the phloem sap related to plant quality, for example the concentrations of nutrients or defensive compounds. *Chaitophorous populicola* aphids migrated to leaves with higher amino acid concentrations within *Populus deltoides* (Gould et al. 2007). Moreover, specialist aphids use specialized metabolites as cues for host finding (Powell et al. 2006). Effector-mediated suppression of plant defenses may also play a role for the choice of feeding sites. Thus, even a few metabolites may influence aphid preferences. Aphid-induced plant volatiles and the honeydew of pre-infesting aphids may also have contributed to aphid choices, while we assume no effect of ear colour, as it was not obviously affected by aphid infestation. In the choice bioassay, we found more nymphs on previously infested ears. This may indicate maternal choice for offspring location. However, it may also be a result of the higher number of adults on these ears, as these probably lay nymphs at their feeding sites and as the nymphs probably stay there due to a low mobility.

This study revealed that *S. avenae* aphids change the composition of the phloem exudates of wheat ears and show higher colony sizes on as well as a preference for previously aphid-infested ears. Thus, *S. avenae* changes its environment in a way improving its fitness, indicating niche construction (Odling-Smee et al. 2003; Trappes 2021). The strength of effects of aphids on plants increases with aphid density (Cabrera et al. 1994; Stewart et al. 2016). A larger previous infestation of *Beta vulgaris* by *M. persicae* led to a better performance of conspecifics (Williams et al. 1998). The colony sizes in our experiment (up to ca. 500 aphids/ ear) were quite high compared to *S. avenae* densities in fields, which did not increase much beyond 50 per tiller (Rabbinge et al. 1979). This may be because we constrained the aphids to one ear, whereas many aphid species in the field start emigration when there are as few as ca. 20 aphids per plant (Hodgson 1991; Rabbinge et al. 1979). The higher aphid densities on the pre-infested plants in our experiment may have caused a positive feedback effect, inducing larger changes in the plant, leading to a further increase of colony sizes. Not many alatae were produced, indicating that the plant quality was appropriate to support aphids even in such high densities. Future studies may shed more light on mechanisms of niche construction, by looking at absolute concentrations of metabolites, identifying more metabolites and studying the effects of the aphid-modulated metabolites on aphids. Moreover, in-depth studies of the probing behavior of *S. avenae* may help to understand the observed differences in aphid performance as well as their choices. There are indications that aphid probing behavior is affected by previous infestation in an aphid species-specific manner (Prado and Tjallingii 1997). Genotypes of *S. avenae* differ in their performance on various host species and preference for host genotypes (De Barro et al. 1995; Zytynska and Preziosi 2011). In contrast to our expectation, the effects of previous aphid infestation on the performance and preference were similar for aphids of a different and of the same clonal lineage. This may indicate that the niche construction mechanisms and niche requirements are similar across genotypes. Future studies should include defined *S. avenae* genotypes to test whether niche construction is genotype-specific and how offspring of different and the same genotype is affected by previous infestation. The implications of a previous infestation with *S. avenae* for competing (aphid) species as well as for antagonists should be also assessed, as these may in turn affect *S. avenae*.

## Supplementary Information

Below is the link to the electronic supplementary material.

Supplementary file1 (DOCX 46 KB)

Supplementary file2 (XLSX 602 KB)

## Data Availability

All data generated or analyzed during this study are included in this published article.
