# Supplementary material for: Niche construction and niche choice by aphids infesting wheat ears
Source: Oecologia. 2024 Sep 3;206(1-2):47–59. doi: 10.1007/s00442-024-05612-0 (PMC11489299; doi:10.1007/s00442-024-05612-0)
Supplement: Supplementary file 1 — Supplementary file1 (DOCX 46 KB) [file 442_2024_5612_MOESM1_ESM.docx]

**Article title: Niche construction and niche choice by aphids infesting wheat ears**

**Journal name: Oecologia**

**Author names: Andreas Bühler, Rabea Schweiger**

**Affiliation and e-mail address of the corresponding author:** Department of Chemical Ecology, Bielefeld University, D-33615 Bielefeld, Germany; rabea.schweiger@uni-bielefeld.de

**SUPPLEMENTARY METHODS 1**

**Metabolic analyses**

The amino acid composition of the phloem exudate samples was determined using high pressure liquid chromatography coupled to fluorescence detection (1260/1290 Infinity, Agilent Technologies, Santa Clara, CA, USA), employing a modified version of the method used in Jakobs and Müller (2018). Derivatization of the amino acids occurred in the autosampler at 6 °C using borate buffer (0.4 M in water, pH = 10.2; Agilent Technologies), ortho-phthaldialdehyde (10 mg ml^-1^ in borate buffer and 3‑mercaptoproprionic acid; Agilent Technologies), 9-fluorenyl-methyl chloroformate (2.5 mg ml^-1^ in acetonitrile; Agilent Technologies) as well as injection diluent [mixture of 100 ml eluent A (see below) and 0.4 ml 85% phosphoric acid (AppliChem)]. Derivatized amino acids were separated on a ZORBAX Eclipse Plus C18 column (250 mm × 4.6 mm, 5 μm particle size, with guard column; Agilent Technologies). The mobile phase was composed of eluent A [1.4 g Na_2_HPO_4_ (≥99%, Carl Roth, Karlsruhe, Germany), 32 mg NaN_3_ (≥98%, Carl Roth) and 3.8 g Na_2_B_4_O_7_ · 10 H_2_O (≥99.5%; Sigma-Aldrich) in 1 l Millipore water, pH adjusted to 8.2 with HCl, filtered through a 0.45 µm membrane] and eluent B [4.5:4.5:1 (v:v:v) methanol (Fisher Scientific), acetonitrile (Fisher Scientific) and Millipore water]. The column temperature was set to 40 °C and samples were separated at a flow rate of 1.5 ml min^‑1^, using 2% eluent B for 0.84 min, followed by a linear gradient to 57% B (reached at 68.4 min) and subsequent column cleaning and equilibration. Amino acid derivatives were detected at an excitation wavelength of 340 nm and an emission wavelength of 450 nm for primary amino acids and 260 nm excitation wavelength and 325 nm emission wavelength for secondary amino acids. Metabolic fingerprinting of polar and semi-polar low-molecular-weight compounds, mainly specialized metabolites, was accomplished with ultra-high performance liquid chromatography (Dionex UltiMate 3000, Thermo Fisher Scientific, San José, USA) coupled to quadrupole time-of-flight mass spectrometry (compact, Bruker Daltonics, Bremen, Germany), using a method modified from Schweiger et al. (2021). The device was equipped with a Kinetex XB-C18 column (150 mm x 2.1 mm, 1.7 µm particle size, with guard column; Phenomenex, Torrance, CA, USA). Chromatographic separation was done at a temperature of 45 °C and a flow rate of 0.5 ml min^-1^. The following gradient involving eluent A [Millipore water with 0.1% formic acid (FA, Fisher Scientific)] and eluent B (acetonitrile with 0.1% FA, Fisher Scientific) was used: 2 to 30% B within 20 min, increase to 75% B within 9 min, followed by cleaning of the column and equilibration. The mass spectrometer was operated in positive electrospray ionization mode. Centroid data were recorded at a spectra rate of 1 Hz in the *m*/*z* (mass-to-charge) range of 50−1300. In the MS mode, we applied an end plate offset of 500 V, a capillary voltage of 4500 V, a nebulizer (N_2_) pressure of 3 bar, a dry gas (N_2_) flow of 12 l min^−1^ at 275 °C, a low mass with an *m*/*z* value of 90, a quadrupole ion energy of 4 eV and a collision energy of 7 eV. For further characterization of putative metabolites, MS/MS spectra for a subset of samples were obtained in AutoMSMS mode. N_2_ was used as collision gas, using increasing isolation widths and collision energies with increasing *m*/*z* of the precursors. Sodium formate was introduced into the ion source prior to each sample for calibration.

**Processing of metabolic data**

Amino acids were identified by comparison of retention times (RT) to those of reference standards using OpenLab ChemStation C.01.07 (Agilent Technologies). They were quantified via the peak areas, divided by the peak areas of the corresponding internal standard. Calibration response factors as well as background subtraction based on the average peak areas in the blanks were applied. Only those amino acids were left in the data set, which occurred in at least half the samples of at least one group (applied separately for the time points: treatments C, I after infestation phase; C, D, S after bioassay phase). Amino acids were categorized as essential for aphids based on literature (Douglas 2006). Metabolic fingerprinting data were analyzed in Data Analysis 4.4 (Bruker Daltonics). The *m*/*z* axis was recalibrated for each sample. Metabolic features, as characterized by specific RT and *m*/*z*, were picked and quantified in MS mode with the T-ReX 3D algorithm in MetaboScape (v. 2021b, Bruker Daltonics). A feature had to occur in at least 2 samples, with an intensity (peak height) threshold of 1000 counts and a minimum compound length of 4 spectra, applying a correlation coefficient threshold of 0.8. Features likely belonging to the same metabolite were sorted together into so-called buckets. For each bucket, the feature with the highest intensity was used for quantification. Any features with RT below 1.2 min were removed, thus excluding metabolites co-eluting within the injection peak. Features which did not have a mean intensity of at least 2000 counts in at least one group (applied separately for the time points: treatments C, I after infestation phase; C, D, S after bioassay phase) or which did not show clear peak shapes were discarded. Peak heights were then divided by the peak heights of the [M+H]^+^ ion of hydrocortisone in the same sample and the mean intensities of the peaks in the corresponding blanks were subtracted. Finally, features which did not occur in at least half the samples of at least one group (applied separately for the time points: treatments C, I after infestation phase; C, D, S after bioassay phase) were discarded. For metabolite identification, an in-house database was used, comparing RT as well as MS spectra and MS/MS spectra.

**TABLE S1** Amino acids in phloem exudates of ears of wheat plants at the end of the infestation phase, in which ears were uninfested (C) or infested by aphids (*Sitobion avenae*, I). The table shows the names and abbreviations (Abbr.) of the amino acids, with the ones being essential for aphids according to Douglas et al. (2006) being listed at the top and underlined. Additionally, the retention times (RT) of the amino acids, their relative concentrations (means ± SD) and the numbers of samples in which they were detected are given. The last columns show the results of pairwise comparisons of the previously aphid-infested ears with the control: Mann-Whitney *U*-tests (*W*-values, *P*-values with correction according to Bonferroni-Holm; in bold if significant at *P* < 0.05) as well as fold changes (based on means; log_2_ scale, indicated in bold if < -1 or > 1).

| **Amino acid** | **Abbr.** | **RT (min)** | **Relative concentrations (mol%)** | |  | **Occurrence** | |  | **I versus C** | |
| --- | --- | --- | --- | --- | --- | --- | --- | --- | --- | --- |
|  |  |  | **No infestation (C)** | **Infestation (I)** |  | **C**  **(out of *n* = 10)** | **I**  **(out of *n* = 20)** |  | **Stats** | **log_2_ fold change** |
| Histidine | HIS | 13.6 | 0.785  ±0.280 | 1.151  ±1.1973 |  | 10 | 20 |  | *W* = 95  *P* = 1 | 0.55 |
| Threonine | THR | 14.7 | 2.835  ±0.232 | 2.684  ±0.517 |  | 10 | 20 |  | *W* = 88  *P* = 1 | -0.08 |
| Valine | VAL | 30.8 | 2.831  ±0.735 | 2.405  ±0.557 |  | 10 | 20 |  | *W* = 72  *P* = 0.692 | -0.24 |
| Methionine | MET | 31.9 | 0.253  ±0.149 | 0.300  ±0.159 |  | 8 | 19 |  | *W* = 98  *P* = 1 | 0.25 |
| Tryptophan | TRP | 35.6 | 0.434  ±0.181 | 0.344  ±0.220 |  | 10 | 19 |  | *W* = 70  *P* = 0.396 | -0.33 |
| Phenylalanine | PHE | 36.7 | 1.106  ±0.255 | 0.959  ±0.365 |  | 10 | 19 |  | *W* = 77  *P* = 0.985 | -0.21 |
| Isoleucine | ILE | 37.3 | 0.835  ±0.162 | 0.717  ±0.245 |  | 10 | 19 |  | *W* = 78  *P* = 1 | -0.22 |
| Leucine | LEU | 39.8 | 1.986  ±0.548 | 1.773  ±0.564 |  | 10 | 20 |  | *W* = 79  *P* = 1 | -0.16 |
| Lysine | LYS | 42.7 | 1.591  ±0.656 | 1.384  ±0.619 |  | 10 | 19 |  | *W* = 94  *P* = 0.812 | -0.20 |
| Aspartic acid | ASP | 2.5 | 26.279  ±6.393 | 25.685  ±6.718 |  | 10 | 20 |  | *W* = 92  *P* = 1 | -0.03 |
| Glutamic acid | GLU | 4.2 | 10.403  ±2.975 | 8.999  ±2.552 |  | 10 | 20 |  | *W* = 68  *P* = 1 | -0.21 |
| Asparagine | ASN | 9.6 | 7.043  ±9.611 | 14.151  ±9.908 |  | 10 | 20 |  | *W* = 42  ***P* = 0.029*** | **1.01** |
| Serine | SER | 10.2 | 8.847  ±1.868 | 8.682  ±1.726 |  | 10 | 20 |  | *W* = 95  *P* = 0.846 | -0.03 |
| Glutamine | GLN | 12.7 | 3.059  ±1.770 | 5.223  ±2.919 |  | 10 | 20 |  | *W* = 47  *P =* 0.057(*) | 0.77 |
| Glycine | GLY | 14.0 | 2.235  ±2.219 | 1.488  ±0.943 |  | 10 | 19 |  | *W* = 87  *P* = 1 | -0.59 |
| Citrulline | CIT | 16.6 | 0.199  ±0.290 | 0.117  ±0.112 |  | 7 | 14 |  | *W* = 94  *P* = 1 | -0.77 |
| β-Alanine | βALA | 17.1 | 0.891  ±1.555 | 0.348  ±0.241 |  | 9 | 19 |  | *W* = 75  *P* = 0.553 | **-1.36** |
| Arginine | ARG | 19.1 | 14.975  ±5.391 | 12.292  ±4.907 |  | 10 | 20 |  | *W* = 69  *P* = 0.549 | -0.28 |
| Alanine | ALA | 19.4 | 0.264  ±0.303 | 0.320  ±0.240 |  | 5 | 14 |  | *W* = 91  *P* = 1 | 0.28 |
| γ-Aminobutyric acid | GABA | 20.1 | 7.809  ±2.223 | 6.902  ±2.911 |  | 10 | 20 |  | *W* = 83  *P* = 0.740 | -0.18 |
| Tyrosine | TYR | 24.6 | 2.551  ±0.466 | 2.477  ±0.831 |  | 10 | 20 |  | *W* = 85  *P* = 1 | -0.04 |
| Proline | PRO | 54.8 | 2.786  ±4.291 | 1.601  ±1.600 |  | 10 | 20 |  | *W* = 79  *P* = 0.931 | -0.80 |

**TABLE S2** Amino acids in phloem exudates of ears of wheat plants at the end of the bioassay phase of the performance bioassay. After ears had been uninfested or infested by aphids (*Sitobion avenae*) in the infestation phase, all ears were infested with aphids in the bioassay phase. The plants that were infested by aphids only in the bioassay phase are indicated with a “C”. For the plants that were infested by aphids both in the infestation phase as well as in the bioassay phase, the aphids in these two phases were either from different clonal lineages (D) or were from the same clonal lineage (S). The table shows the names and abbreviations (Abbr.) of the amino acids, with the ones being essential for aphids according to Douglas et al. (2006) being listed at the top and underlined. Additionally, the retention times (RT) of the amino acids, their relative concentrations (means ± SD) and the numbers of samples in which they were detected are given. The last columns show the results of pairwise comparisons between the groups (D versus C, S versus C): Mann-Whitney *U*-tests (*W*-values; *P*-values with correction according to Bonferroni-Holm; in bold if significant at *P* < 0.05) as well as fold changes (based on the means; log_2_ scale, indicated in bold if < -1 or > 1).

| **Amino acid** | **Abbr.** | **RT (min)** | **Relative concentrations (mol%)** | | |  | **Occurrence** | | |  | **D versus C** | |  | **S versus C** | |
| --- | --- | --- | --- | --- | --- | --- | --- | --- | --- | --- | --- | --- | --- | --- | --- |
|  |  |  | **C** | **D** | **S** |  | **C**  **(out of *n* = 16)** | **D**  **(out of *n* = 10)** | **S**  **(out of *n* = 17)** |  | **Stats** | **log_2_ fold change** |  | **Stats** | **log_2_ fold change** |
| Histidine | HIS | 13.6 | 2.053  ±1.296 | 1.850  ±0.855 | 2.025  ±1.058 |  | 16 | 10 | 17 |  | *W* = 79  *P* = 1 | -0.15 |  | *W* = 132  *P* = 1 | -0.02 |
| Threonine | THR | 14.7 | 3.213  ±0.977 | 3.077  ±0.945 | 3.154  ±0.990 |  | 16 | 10 | 17 |  | *W* = 81  *P* = 1 | -0.06 |  | *W* = 132  *P* = 1 | -0.03 |
| Valine | VAL | 30.8 | 3.640  ±0.811 | 3.553  ±0.433 | 3.605  ±0.630 |  | 16 | 10 | 17 |  | *W* = 80  *P* = 1 | -0.04 |  | *W* = 127  *P* = 1 | -0.01 |
| Methionine | MET | 31.9 | 0.862  ±0.488 | 0.588  ±0.517 | 0.686  ±0.701 |  | 11 | 7 | 11 |  | *W* = 86.5  *P* = 1 | -0.55 |  | *W* = 137  *P* = 1 | -0.33 |
| Tryptophan | TRP | 35.6 | 0.488  ±0.384 | 0.568  ±0.354 | 0.913  ±0.761 |  | 11 | 8 | 14 |  | *W* = 68  *P* = 0.541 | 0.21 |  | *W* = 81.5  *P* = 0.150 | 0.90 |
| Phenylalanine | PHE | 36.7 | 1.998  ±0.446 | 1.884  ±0.397 | 1.943  ±0.714 |  | 16 | 10 | 16 |  | *W* = 94  *P* = 0.985 | -0.08 |  | *W* = 136  *P* = 1 | -0.04 |
| Isoleucine | ILE | 37.3 | 1.373  ±0.456 | 1.439  ±0.249 | 1.090  ±0.785 |  | 15 | 10 | 12 |  | *W* = 72  *P* = 1 | 0.07 |  | *W* = 118.5  *P* = 1 | -0.33 |
| Leucine | LEU | 39.8 | 2.750  ±1.373 | 2.769  ±0.541 | 2.686  ±0.990 |  | 15 | 10 | 16 |  | *W* = 85  *P* = 1 | 0.01 |  | *W* = 135.5  *P* = 1 | -0.03 |
| Lysine | LYS | 42.7 | 1.373  ±0.930 | 1.771  ±0.265 | 1.791  ±0.587 |  | 12 | 10 | 16 |  | *W* = 56  *P* = 0.429 | 0.37 |  | *W* = 87  *P* = 0.240 | 0.38 |
| Aspartic acid | ASP | 2.5 | 31.551  ±7.953 | 32.714  ±4.155 | 29.121  ±8.874 |  | 16 | 10 | 17 |  | *W* = 82  *P* = 1 | 0.05 |  | *W* = 105  *P* = 1 | -0.12 |
| Glutamic acid | GLU | 4.2 | 10.670  ±5.466 | 9.226  ±4.445 | 7.823  ±3.869 |  | 16 | 10 | 17 |  | *W* = 92  *P* = 1 | -0.21 |  | *W* = 91  *P* = 0.828 | -0.45 |
| Asparagine | ASN | 9.6 | 4.289  ±1.153 | 4.388  ±1.594 | 5.358  ±2.707 |  | 16 | 10 | 17 |  | *W* = 81  P = 0.979 | 0.03 |  | *W* = 107  *P* = 0.618 | 0.32 |
| Serine | SER | 10.2 | 10.230  ±8.329 | 11.405  ±8.577 | 12.156  ±7.102 |  | 16 | 10 | 17 |  | *W* = 61  *P* = 0.336 | 0.16 |  | *W* = 91  *P* = 0.110 | 0.25 |
| Glutamine | GLN | 12.7 | 7.081  ±4.606 | 6.872  ±3.986 | 10.008  ±11.042 |  | 16 | 10 | 17 |  | *W* = 78  *P* = 0.975 | -0.04 |  | *W* = 116  *P* = 0.975 | 0.50 |
| Glycine | GLY | 14.0 | 3.359  ±3.926 | 3.511  ±2.182 | 3.109  ±3.359 |  | 14 | 9 | 12 |  | *W* = 75  *P* = 1 | -0.17 |  | *W* = 120  *P* = 1 | -0.35 |
| Citrulline | CIT | 16.6 | 0.989  ±1.402 | 0.657  ±0.587 | 0.852  ±1.043 |  | 11 | 8 | 14 |  | *W* = 76  *P* = 1 | -0.59 |  | *W* = 134.5  *P* = 1 | -0.21 |
| β-Alanine | βALA | 17.1 | 0.014  ±0.056 | 0.013  ±0.041 | 0.141  ±0.464 |  | 1 | 1 | 4 |  | *W* = 77.5  *P* = 0.820 | -0.12 |  | *W* = 112.5  *P* = 0.553 | **3.33** |
| Arginine | ARG | 19.1 | 6.089  ±1.442 | 6.421  ±0.914 | 6.537  ±1.340 |  | 16 | 10 | 17 |  | *W* = 76  *P* = 1 | 0.08 |  | *W* = 122  *P* = 1 | 0.10 |
| Alanine | ALA | 19.4 | 0.740  ±0.457 | 0.738  ±0.311 | 0.879  ±0.372 |  | 14 | 9 | 17 |  | *W* = 75  *P* = 1 | 0.25 |  | *W* = 110  *P* = 1 | 0.25 |
| γ-Aminobutyric acid | GABA | 20.1 | 2.767  ±1.896 | 2.048  ±1.444 | 1.455  ±0.896 |  | 15 | 9 | 16 |  | *W* = 97.5  *P* = 0.740 | -0.93 |  | *W* = 75.5  *P* = 0.092(*) | -0.93 |
| Tyrosine | TYR | 24.6 | 2.309  ±1.267 | 2.508  ±1.014 | 2.957  ±1.543 |  | 15 | 10 | 17 |  | *W* = 60  *P* = 1 | 0.36 |  | *W* = 105  *P* = 0.828 | 0.36 |
| Proline | PRO | 54.8 | 1.561  ±1.038 | 2.001  ±1.021 | 1.712  ±1.277 |  | 16 | 10 | 16 |  | *W* = 60  *P* = 0.93 | 0.13 |  | *W* = 134  *P* = 0.958 | 0.13 |
